# A Review of NIDDK‐Funded Studies of Urological Chronic Pain Conditions

**DOI:** 10.1002/nau.70223

**Published:** 2026-01-28

**Authors:** J. Quentin Clemens

**Affiliations:** ^1^ Department of Urology University of Michigan, 1500 East Medical Center Drive, Taubman Center 3875 Ann Arbor Michigan USA

## Abstract

**Introduction:**

This review highlights NIDDK‐funded clinical research studies focused on interstitial cystitis/bladder pain syndrome (IC/BPS) and chronic prostatitis/chronic pelvic pain syndrome (CP/CPPS).

**Methods:**

Since 1987, the NIDDK has funded numerous cohort studies, epidemiologic studies, and clinical trials for these conditions.

**Results:**

While the majority of clinical trials have not demonstrated positive results, these research efforts have provided essential information about these conditions, which have informed clinical practice guidelines and enhanced the clinical care of patients.

**Conclusions:**

The data from these studies are a valuable resource that is available via the NIDDK Data Repository for future analysis.

Over the past 50 years, the NIDDK has devoted substantial resources to studying urological chronic pain conditions, with the majority of studies focusing on interstitial cystitis/bladder pain syndrome (IC/BPS) or chronic prostatitis/chronic pelvic pain syndrome (CP/CPPS). In the 1980s, these conditions were poorly understood and were considered rare and largely psychogenic in nature. Consequently, there was little interest in studying them. In 1984, the Interstitial Cystitis Association (ICA) was founded by Vicki Ratner, MD (an IC/BPS patient and orthopedic surgeon). Advocacy efforts by the ICA and others led to recognition of IC/BPS and CP/CPPS as “legitimate” medical conditions with substantial impact on patients. These efforts also resulted in increased funding opportunities for these conditions. The NIDDK research strategy evolved in a similar fashion for both IC/BPS and CP/CPPS, and is outlined in Table 1.

**Table 1:** Timeline for NIDDK‐funded clinical studies of IC/BPS and CP/CPPS.

IC/BPS	CP/CPPS
NIDDK Consensus Definition 1987, 1990	NIDDK Consensus Definition 1995
ICDB Cohort Study 1991−1996	CPC Cohort Study 1997−2002
Validated Questionnaire (Interstitial Cystitis Symptom Index) 1997	Validated Questionnaire (Chronic Prostatitis Symptom Index) 1997−2002
Clinical Trials (ICCRN 1) 1998−2003	Clinical Trials (CPCRN 1) 1997−2002
More Clinical Trials (ICCRN 2) 2003−2008	More Clinical Trials (CPCRN 2) 2003−2008
Cohort Study (MAPP) 2008−2023	

## 1 Cohort Studies

Initial studies included the Interstitial Cystitis Database (ICDB) study for IC/BPS [1] and the Chronic Prostatitis Cohort (CPC) study for CP/CPPS [2]. These two cohort studies developed standardized definitions and validated outcome measures for IC/BPS and CP/CPPS and provided valuable information about clinical characteristics and disease course over time. Importantly, these efforts demonstrated that it was possible to identify and recruit well‐defined groups of patients and track their symptoms with standardized outcome measures. This made it possible to test therapies in subsequent randomized controlled trials (RCTs).

### 1.1 Clinical Trials for IC/BPS

The Interstitial Cystitis Collaborative Research Network (ICCRN) conducted four RCTs in patients with IC/BPS. Unfortunately, none of these studies demonstrated efficacy for the tested therapies when compared with placebo/sham. In the initial trial, participants were randomized to receive: (1) oral pentosan polysulfate, (2) oral hydroxyzine, (3) both medications, or (4) placebo [3]. After 24 weeks of treatment, there were no significant differences observed in the active treatments when compared with placebo. Recruitment was found to be difficult for this study since these agents were readily available via prescription, and many patients were unwilling to potentially receive a placebo. The second trial compared 6 weekly instillations of intravesical bacillus Calmette‐Guerin (BCG) to sham bladder instillation [4]. While there were substantial uncontrolled data suggesting efficacy of BCG, when subjected to a controlled trial, it was found to be no more effective than the sham therapy in reducing IC/BPS symptoms at 34 weeks. The third trial compared oral amitriptyline to placebo in patients who were naive to this therapy [5]. Study participants in both treatment arms also received a standardized education and behavioral modification program. The drug dose was increased over a 6‐week period from 10 mg up to 75 mg once daily. At 12 weeks, there was no difference in outcomes between the two groups. However, in the subset of participants who were able to tolerate the higher doses (50 or 75 mg), there was a statistically significant symptom improvement when compared with placebo. The fourth trial intended to compare the immunosuppressant mycophenolate mofetil to a placebo in patients with treatment‐refractory IC/BPS [6]. However, the trial was halted after a new black box warning was issued by the drug manufacturer. Interim analysis of the data identified an observed reduced efficacy of mycophenolate mofetil compared to placebo. Therefore, the study was terminated.

## 2 Clinical Trials for CP/CPPS

The Chronic Prostatitis Collaborative Research Network conducted three RCTs in men with CP/CPPS. Similar to the IC/BPS trials, none of the tested therapies demonstrated efficacy for the pre‐specified primary outcomes. In the first trial, participants were randomized to receive: (1) oral tamsulosin 0.4 mg daily, (2) oral ciprofloxacin 500 mg twice daily, (3) a combination of the two drugs, or (4) placebo [7]. After 6 weeks of therapy, no significant differences in outcomes were observed across the treatment groups. It was noted that most study participants had long‐standing symptoms, and therefore, it was likely that many had previously failed treatment with these medications. To address these concerns, a second trial was conducted, which compared a different alpha‐blocker (alfuzosin 10 mg daily) with a placebo [8]. Notably, this study enrolled men with CP/CPPS who had not previously been treated with an alpha‐blocker and who reported having had symptoms for 2 years or less. Despite these adjustments to the study inclusion criteria, the alfuzosin group demonstrated no significant differences in outcomes compared with placebo after 12 weeks of therapy. In the third trial, men with CP/CPPS were randomly assigned to receive pregabalin or placebo in a 2:1 ratio and were treated for 6 weeks [9]. Pregabalin dosage was increased from 150 to 600 mg/day during the first 4 weeks. After 6 weeks of therapy, there was no difference between the two groups in the primary endpoint. However, participants who received pregabalin demonstrated statistically significant improvements in most secondary endpoints when compared with placebo.

### 2.1 Urologic Pelvic Pain Collaborative Research Network (UCCRN)

The ICCRN and CPCRN then combined efforts to conduct a clinical trial to examine the efficacy of pelvic floor physical therapy (PFPT) in both men and women. After the PFPT intervention had been standardized across sites, two pilot studies were conducted (one for men with CP/CPPS and one for women with IC/BPS) [10]. Patients were required to have pelvic floor muscle tenderness on examination in order to be eligible for the study. The treatment groups received connective tissue manipulation and trigger point release, including transvaginal/transrectal treatment of the pelvic soft tissues. Control groups received a full‐body therapeutic massage. Each treatment arm consisted of 10 weekly treatment sessions. This initial pilot study demonstrated substantial efficacy for the PFPT arm in both men and women, but men experienced an equivalent symptom reduction following the control therapeutic massage. These pilot results were used to develop an adequately powered full‐scale trial in women with IC/BPS [11], which found that a significantly higher proportion responded to treatment with PFPT (59%) than with therapeutic massage (26%).

### 2.2 Epidemiologic Studies

The NIDDK also funded several epidemiologic studies that provided information about incidence, prevalence, comorbidities, quality of life impact, and costs of IC/BPS and CP/CPPS [12, 13, 14, 15, 16]. From these studies, it became evident that urologic pain conditions were more common than previously thought, and that many patients with IC/BPS and CP/CPPS were diagnosed with other chronic pain conditions (e.g., irritable bowel syndrome, fibromyalgia, migraines, vulvodynia, etc.).

### 2.3 Basic Science Studies

In addition to the clinical studies summarized above, numerous basic science grants have been awarded by the NIDDK to investigate potential pathophysiologic mechanisms responsible for IC/BPS and CP/CPPS. These included both in vivo animal model studies and in vitro studies. A detailed review of these investigations is outside the scope of this review article. Despite these extraordinary efforts, no consensus agreement has been achieved on an underlying etiology for IC/BPS or CP/CPPS, and no disease biomarkers have been identified.

### 2.4 Multidisciplinary Approach to the Study of Chronic Pelvic Pain (MAPP) Cohort Study

The limitations of previous studies and results suggesting associations with other chronic pain conditions prompted the NIDDK to initiate a new research program for the study of IC/BPS and CP/CPPS, the MAPP Research Network [17]. The premise of the MAPP network was that the traditional bladder and prostate centered focus of IC/BPS and CP/CPPS (collectively referred to as Urologic Chronic Pelvic Pain Syndrome, or UCPPS) should be broadened to a systemic view of disease in which the interplay between the genitourinary system and other physiological systems (e.g., the central nervous system), is highlighted. In addition, it was proposed that studies of UCPPS would benefit from incorporating broad approaches involving a diversity of urologic and non‐urologic disciplines to promote a more comprehensive characterization of patient phenotypes. The MAPP network adopted a highly collaborative and integrated research strategy that involved investigators representing traditional urologic disciplines, as well as non‐urologic expertise, including experts in pain research, neurobiology and neuroimaging, infectious disease, biomarker discovery, animal modeling, epidemiology, and psychology, among many others. The overarching goal of the MAPP Research Network was to provide findings that would be useful for designing future clinical trials and ultimately to improve the clinical management of UCPPS patients.

The foundation for the MAPP network included two prospective cohort studies. The Epidemiology/Phenotyping Study (EPS) was conducted from 2008 to 2014 and followed 424 UCPPS patients for 12 months, with biweekly internet‐based questionnaires [18]. In addition, in‐person “deep phenotyping” studies were conducted at baseline, 6 and 12 months. The “deep phenotyping” assessments included a series of questionnaires (sociodemographics, medical history, urologic symptoms, non‐urologic symptoms, psychosocial symptoms), standardized physical examination, and biospecimen collection (urine, plasma, and cheek swab DNA). An additional 380 healthy controls matched on sex and age, and 190 “positive” controls (with other chronic pain disorders such as fibromyalgia, irritable bowel syndrome, or chronic fatigue syndrome) were also recruited and phenotyped at baseline. A subset of EPS participants underwent experimental quantitative sensory testing (QST) and functional magnetic resonance imaging. The second MAPP cohort study was termed the Symptom Patterns Study and was conducted from 2014 to 2022 [19]. The SPS extended the MAPP I EPS cohort study, including expanded longitudinal measures, a run‐in period, and a 36‐month longitudinal follow‐up period. The SPS recruited 620 UCPPS participants who completed in‐person “deep phenotyping” visits at baseline, 6, 18, and 36 months, as well as internet‐based questionnaires every 3 months. The SPS also included weekly “run‐in” assessments X4 at baseline to determine symptom stability. In addition, 72 Controls without UCPPS were recruited and completed baseline and 6‐month in‐clinic “deep phenotyping” assessments. Notably, multiple neuroimaging scans, QST measures, and biospecimens were obtained on all SPS participants throughout the 3‐year time period, providing a very rich dataset for analysis.

Major MAPP findings have been summarized elsewhere [20, 21]. MAPP studies showed that UCPPS pain and urinary symptoms covary and should be evaluated separately. Approximately 30% of patients reported improvement in pain and/or urinary symptoms over 3 years. Participants with UCPPS reported more psychosocial difficulties than healthy controls, and poor psychosocial functioning was associated with a low likelihood of symptom improvement over time. Longitudinal clinical changes in UCPPS correlated with structural and functional brain changes, and many patients experienced global multisensory hypersensitivity. Additionally, UCPPS symptom profiles were distinguishable by biological correlates, such as immune factors. Symptom flares were common and differed considerably in intensity, duration, and influence on quality of life. Participants with UCPPS who reported widespread pain (i.e., pain beyond the pelvis) had more severe UCPPS symptoms and less likelihood of symptom improvement than those with pelvic pain only. Importantly, the presence of widespread pain appears to predict treatment response, with “systemic” therapies demonstrating better efficacy in patients with widespread pain [22, 23].

In conclusion, the NIDDK has funded numerous studies of IC/BPS and CP/CPPS, which have greatly improved our knowledge of these conditions and have informed clinical practice guidelines and enhanced the clinical care of patients. The data from these studies are a valuable resource that is available via the NIDDK Data Repository for future analysis.

## Funding

The author received no specific funding for this work.

## Ethics Statement

The author has nothing to report.

## Consent

The author has nothing to report.

## Conflicts of Interest

The author declares no conflicts of interest.

## Data Availability

The author has nothing to report.
